# High and Low Doses of Ionizing Radiation Induce Different Secretome Profiles in a Human Skin Model

**DOI:** 10.1371/journal.pone.0092332

**Published:** 2014-03-18

**Authors:** Qibin Zhang, Melissa Matzke, Athena A. Schepmoes, Ronald J. Moore, Bobbie-Jo Webb-Robertson, Zeping Hu, Matthew E. Monroe, Wei-Jun Qian, Richard D. Smith, William F. Morgan

**Affiliations:** 1 Biological Sciences Division, Pacific Northwest National Laboratory, Richland, Washington, United States of America; 2 Computational Biology and Bioinformatics, Pacific Northwest National Laboratory, Richland, Washington, United States of America; Northwestern University Feinberg School of Medicine, United States of America

## Abstract

It is postulated that secreted soluble factors are important contributors of bystander effect and adaptive responses observed in low dose ionizing radiation. Using multidimensional liquid chromatography-mass spectrometry based proteomics, we quantified the changes of skin tissue secretome – the proteins secreted from a full thickness, reconstituted 3-dimensional skin tissue model 48 hr after exposure to 3, 10 and 200 cGy of X-rays. Overall, 135 proteins showed statistical significant difference between the sham (0 cGy) and any of the irradiated groups (3, 10 or 200 cGy) on the basis of Dunnett adjusted t-test; among these, 97 proteins showed a trend of downregulation and 9 proteins showed a trend of upregulation with increasing radiation dose. In addition, there were 21 and 8 proteins observed to have irregular trends with the 10 cGy irradiated group either having the highest or the lowest level among all three radiated doses. Moreover, two proteins, carboxypeptidase E and ubiquitin carboxyl-terminal hydrolase isozyme L1 were sensitive to ionizing radiation, but relatively independent of radiation dose. Conversely, proteasome activator complex subunit 2 protein appeared to be sensitive to the dose of radiation, as rapid upregulation of this protein was observed when radiation doses were increased from 3, to 10 or 200 cGy. These results suggest that different mechanisms of action exist at the secretome level for low and high doses of ionizing radiation.

## Introduction

Both natural and man-made sources of ionizing radiation contribute to human exposure and consequently pose a possible risk to human health. While natural background radiation is unavoidable, the increased medical use of radiation, e.g. X-ray and CT scans for diagnosis, inevitably increases the public's health risk concerns over exposure of low doses of ionizing radiation.[1], [2] Traditionally, ionizing radiation-induced cancer risks at low doses have been extrapolated from high dose studies, for which the effects on human health and biological systems are well documented, based on a linear no threshold dose-response model between radiation dose and effects;[3]–[5] however, recent literature suggests that the mechanisms of action for many biological endpoints may be different at low doses from those observed at high doses.[6]–[11]

Secreted proteins play key roles in cell–cell signalling, communication, migration and growth, as they reflect the various stages of biological and pathological conditions.[12] Secreted soluble factors have long been postulated as the contributor of bystander effects observed from both *in vitro* and *in vivo* studies following ionizing radiation, where detrimental health effects of radiation exposure occur on neighbouring cells of the irradiated cells.[13] Many past studies on secreted factors were focused on a limited number of known proteins using targeted biochemical assays of Western-Blot, ELISA or protein microarrays;[14], [15] despite decades of study, the identities of these secreted factors remain elusive.

Using advanced LC-MS based proteomics technologies, we now present a comprehensive profiling of proteins secreted into culturing medium by a model 3D human skin tissue upon various doses of X-ray irradiation. Proteomic coverage of skin secretome was broadened by removal of medium proteins and extensive fractionation at peptide level before LC-MS/MS analysis. Quantitative comparison between low (3, 10 cGy) and high (200 cGy) doses of ionizing radiation indicates that different action mechanisms exist between low and high doses.

## Materials and Methods

### Tissue Culture and Irradiation

The human skin tissues, EpiDerm Full Thickness 400, were purchased from the MatTek Corporation (Ashland, MA). Upon arrival, the tissues were randomized into 6-well plates, and cultured in EFT-400-MM maintenance medium (Dulbecco's modified Eagle's medium supplemented with epidermal growth factor, insulin, hydrocortisone and other proprietary stimulators of epidermal differentiation) that also obtained from the MatTek Corporation. The tissues were grown in an atmosphere of 5% CO_2_ at 37°C and given 5 mL fresh medium every 2 days. Radiation experiments were performed after the tissues were acclimated for 5–7 days in our laboratory.

The tissues were randomized into four radiation dose groups (0, 3, 10 and 200 cGy) with twelve tissues (n = 12) per group. The irradiation of tissues with Low-LET (2 keV/μm) X-ray was carried out using a Pantak XRAD 320 irradiator (Precision X-Ray, North Branford, CT) operated at 300 kV. The dose rates were as follows: 3 and 10 cGy at 6 cGy/min, 200 cGy at 60 cGy/min. Following irradiation, tissues were returned to the same atmosphere as before collection at 37°C. At 48 hr post-irradiation, the media were collected and snap-frozen in liquid nitrogen and stored at −80°C until further processing.

### Proteomic Sample Preparation

Aliquots of each medium sample were pooled according to the radiation dose group, then 6 mL of the pooled samples and 1.5 mL of each individual sample were subjected to immunoaffinity depletion to remove albumin and other abundant proteins using an IgY-14 LC5 column (Sigma-Aldrich) coupled with an Agilent 1100 series HPLC, under the conditions suggested by the manufacture for mobile phases and flow rate. The flow-through fractions (low abundance proteins) were collected and concentrated in Amicon Ultra-15 concentrators (Millipore) with MWCO of 3 kDa, followed by a buffer exchange to 50 mM NH_4_HCO_3_ in the same unit according to the manufacturer's instructions. Sample proteins were next sequentially denatured with 8 M urea, reduced with dithiothreitol, alkylated with iodoacetamide, and digested with trypsin (Promega) at a trypsin/protein ratio of 1∶50; the peptide mixtures were then cleaned with C_18_ SPE cartridges (Sigma-Aldrich) and dried *in vacuo* before reconstituted with 0.1% formic acid for LC-MS analysis.

### Instrument Analysis

For the pooled samples, protein digests were on-line fractioned and further separated using a custom-built 2D-LC system. The 2D-LC system was composed of two Agilent 1200 nanoflow pumps and one 1200 capillary pump (Agilent Technologies), various Valco valves (Valco Instruments Co., Houston, TX) and a PAL autosampler (Leap Technologies, Carrboro, NC). Full automation was made possible by custom software that allows for parallel event coordination and therefore near 100% MS duty cycle through the use of one strong cation exchange (SCX) column (15 cm×150μm, 5μ PolySulfoethyl A particles, Poly LC Inc.) in the 1^st^ dimension, and two trapping columns (4 cm×100μm, 3.6μ Aries C_18_ particles, Phenomenex) and two analytical columns (35 cm×75μm, 3μ Jupiter C_18_ particles, Phenomenex) in the 2^nd^ dimension. All columns were manufactured in-house by slurry packing of particles into fused silica (Polymicro Technologies Inc., Phoenix, AZ) using a 1 cm sol-gel frit for particle retention. Mobil phases used in the 1^st^ dimension were 0.05% acetonitrile in water and 500 mM ammonium formate to fractionate the samples (injection amount 9 μg) into 15 fractions; and 0.1% formic acid in water and 0.1% formic acid in acetonitrile were used as mobile phases in the 2^nd^ dimension for reversed phase separation of the 15 peptide fractions with a gradient length of 100 min. The eluents from the 2^nd^ dimension separation were electrospray ionized and analyzed by an Orbitrap Velos Pro hybrid mass spectrometer (ThermoScientific), which was operated in data-dependent MS/MS mode, with one high resolution MS scan followed by 10 low resolution CID MS/MS scan events.

For each individual sample, protein digests were separated on a 4-column custom-built capillary LC system similar as reported previously.[16] Reversed-phase separations were carried out on 35 cm × 75 μm i.d. fused silica columns packed in house using 3μ Jupiter C_18_ particles (Phenomenex). Mobile phases consisted of 0.1% formic acid in water (A) and 0.1% formic acid in 100% acetonitrile (B) with a 100 min gradient. MS analyses were performed using an Exactive Orbitrap mass spectrometer (ThermoScientific) outfitted with a custom electrospray ionization (ESI) interface. MS spectra (AGC 3 × 10^6^) were collected in positive ionization mode from 400 to 2000 *m*/*z* at a resolution of 100 k. Triplicate analyses were performed on each individual sample.

### Data Analysis

Identification and quantification of peptides was performed using the accurate mass and time (AMT) tag approach.[17] The datasets acquired in the 2D LC-MS/MS analyses were searched using the SEQUEST algorithm to match the MS/MS fragment spectra with sequences from the UniProt human protein database (2010-05-05 version with 20,276 protein entries) with static carbamidomethylation of cysteine used as the modification. DeconMSN was used to accurately determine the parent ion monoisotopic mass.[18] Database-matched results were filtered using a very rigorous MSGF cut-off score of 1 E-12,[19] which resulted in an estimated false discovery rate of 0.3% at the unique peptide level in a decoy database search. The list of confidently identified peptides was populated into an AMT tag database. The peptide elution times from each LC-MS/MS analysis were normalized to a range of 0 to 1 using a predictive normalized elution time (NET) model.[20] Both calculated monoisotopic masses and observed NETs of identified peptides were included in the AMT tag database. For the high resolution LC-MS datasets obtained from individual study samples, Decon2LS was used for peak finding and deisotoping at the individual MS spectrum level.[21] The output of Decon2LS that contains features of monoisotopic mass, intensity of most abundant isotope and elution time were further processed with VIPER to cluster mass and chromatographic elution features across datasets,[22] and to match the clustered features with the AMT tag database to identify the peptides and generate abundance profiles. Uniqueness probability (UP) score of >0.5 and an FDR of <10% were used for each dataset, and 1 ppm mass measurement error in at least 1 dataset were used to remove the ambiguities in database matching and to improve the subsequent identification and quantification of clustered features.

Crosstab file that contains peptide ID and peptide abundance in each sample was exported to MatLab® for statistical analysis. This includes quality control, normalization, protein roll-up, and peptide and protein level comparative statistical analyses. Peptide abundances were transformed to the log_10_ scale. Missing data values were not imputed. Quality control processing was performed to identify and remove contaminant proteins, redundant peptides, peptides with an insufficient amount of data across the set of samples,[23] and LC-MS runs that showed significant deviation from the standard behavior of all LC-MS analyses.[24] Peptides were normalized using a statistical procedure for the analysis of proteomic normalization strategies (SPANS) that identifies the peptide selection method and data scaling factor which introduces the least amount of bias into the dataset.[25] The peptide abundance values were normalized across the technical replicates with a peptide subset based on the L-order statistic using a median centering of the data. Normalized log_10_ abundance values of peptides were averaged across the technical replicates within each biological sample, and evaluated with a Dunnett adjusted 2-sided t-test to identify quantitative significance patterns in the peptide data. For protein quantification, peptide level significance patterns based on the comparisons of the 3, 10 and 200 cGy groups to the control group (0 cGy) were used to select appropriate peptides for protein roll-up, which was based on the down-selection of peptides that have a common statistical trend followed by a standard average to a reference peptide, where the reference peptide was defined as the peptide with the least amount of missing data. Similarly, a Dunnett adjusted 2-sided t-test was used to assess differences in protein average abundance between the control group (0 cGy) and the 3, 10 and 200 cGy irradiated groups. Dunnett test p-values less than 0.05 were deemed statistically significant. The Uniprot data base (www.uniprot.org) was used to search the subcellular location of each significantly changed protein.

### Ingenuity Pathway Analysis

To identify potential radiation-perturbed molecular pathways in the skin secretome, Ingenuity pathway analysis (IPA, www.ingenuity.com) was applied to relate the differentially expressed proteins to each other based on their interaction and function against a high-quality expert-curated knowledge database derived from the literature. In brief, all proteins that were statistically significantly expressed along with their fold change (log2 ratio) and t-test values with respect to 0 cGy group were uploaded into the IPA software, which extracted the overlapping networks among the candidate proteins from the curated database. Comparison analyses were made on both Functions and Canonical Pathways levels between different radiation doses, along with a score representing the log probability of a particular network being found by random chance. The *p* values were calculated using right-tailed Fisher's exact tests.

## Results and Discussion

### Overview of sample preparation, data acquisition and data processing

Identification of secreted proteins from culturing medium presents significant challenge due to the composition of conditioned medium. SDS-PAGE gel analysis showed that significant amount of albumin existed in the medium, therefore we used immunodepletion to remove the highly abundant medium-composing proteins in order to broaden the coverage of the proteome secreted by the skin. Depletion of highly abundant proteins using avian polyclonal IgY antibody columns has demonstrated excellent reproducibility.[26] Therefore, all of the medium samples in this study were depleted to enrich the low abundant secreted proteins using an IgY-14 column.

To overcome the under-sampling issue and increase throughput in LC-MS based global proteomics analysis, we applied AMT tag strategy for the label free quantitation of each individual sample.[17] An on-line 2D LC-MS/MS system was used to fractionate and analyze the peptide digests obtained from 4 samples pooled from each radiation dose group. The peptides identified from these LC-MS/MS analyses served as the basis for building the skin secretome AMT peptide database. The final AMT tag database contained 10,149 peptides from 1547 human proteins available for matching to the high resolution LC-MS datasets acquired from the individual irradiated media samples.

After matching the individual sample LC-MS dataset with the AMT tag database, 5171 unique peptides were identified and quantified from the 48 individual samples. After quality control processing to remove outlier LC-MS runs, redundant peptides, and peptides with missing data,[23], [24] 3386 peptides were retained for further downstream data analysis, which include a proteomic normalization strategy to select peptide and data scaling factor that introduces the least amount of bias into the dataset,[25] and comparative statistical analysis was done on the log transformed abundance values for each retained peptide to calculate the statistical trend, as detailed in the Methods Section. Protein roll-up was based on the down-selection of peptides that have a common statistical trend followed by a standard average to a reference peptide, after filtering the peptides that were redundant, had low data content, or were outside the dominant significance pattern calculated on peptide level. Trends were based on the statistical significance indicator variables devised from statistical tests performed on the peptides. The final protein quantification was based upon a standard roll-up to a reference peptide, where the reference peptide was defined as the peptide with the least amount of missing data. In total, 880 human proteins were rolled up from 2,229 peptides, and differences in protein average abundance between different radiation conditions were assessed using a Dunnett adjusted 2-sided t-test to compare control group with the radiated groups of 3, 10 and 200 cGy. Dunnett p-values less than 0.05 were deemed statistically significant.

### Statistical analysis showing dose dependent perturbation of secretome by ionizing radiation

**Figure 1:**
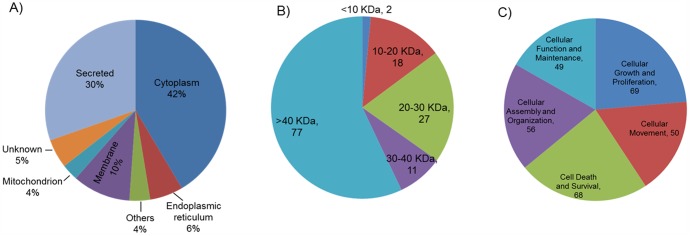
Overview of the A) subcellular location, B) molecular size distribution and C) molecular and cellular functions of the 135 proteins that showed significant variation between irradiated and sham skin tissues. Others: subcellular location known, but not being included in the main categories; Unknown: subcellular location unknown. Protein may have more than one function.

Overall, 135 proteins showed a statistical significant difference between the sham (0 cGy) and any of the irradiated groups (3, 10 or 200 cGy) on the basis of Dunnett adjusted t-test. Thirty three proteins with the largest fold change (>1.5 fold for up- and <2.0 for downregulation) are listed in Table 1, while the details of all proteins are in the Supporting Information (Table S1). To facilitate categorization of these 135 proteins, the Uniprot protein database was searched to identify their subcellular locations with relatively broad definition of subcellular location terms. As shown in Figure 1A, majority of these proteins were either originated from the cytoplasm (42%) or directly secreted into the extracellular matrix (30%), suggesting the quality of our sample preparation and analysis process, as well as the potential effects of ionizing radiation to the cellular processes. The size distribution of these proteins is shown in Figure 1B. Functional analysis using IPA showed that these proteins were related with protein binding, cell signalling, cell movement, epidermis development, endoplasmic reticulum stress response, inflammation, free radical scavenging, etc. The number of proteins represented by major molecular and cellular functions is shown in Figure 1C.

**Table 1:** Selected list of statistically significant proteins secreted into the medium by a full thickness skin tissue model at different doses (3, 10 and 200 cGy) of X-rays when compared with sham controls (0 cGy).

	Group Differences (log)			DUNNETT Adjusted P-Values			DUNNETT TRENDS					
PROTEIN	3 vs. 0	10 vs. 0	200 vs. 0	3 vs. 0	10 vs. 0	200 vs. 0	3 vs. 0	10 vs. 0	200 vs. 0	TREND	SUBCELLULAR LOCATION	Main Biological Function
1433B_HUMAN	0.139	−0.109	−1.081	0.9622	0.9806	0.0115	0	0	−1	down	cytoplasm	signaling pathway regulation
1433E_HUMAN	−0.074	−0.216	−1.214	0.9944	0.8896	0.0060	0	0	−1	down	cytoplasm	signaling pathway regulation
1433S_HUMAN	0.063	−0.296	−1.170	0.9970	0.7914	0.0126	0	0	−1	down	secreted	signaling pathway regulation
1433T_HUMAN	0.030	−0.330	−1.238	0.9996	0.6990	0.0044	0	0	−1	down	cytoplasm	signaling pathway regulation
A4_HUMAN	0.410	0.727	0.619	0.2097	0.0097	0.0315	0	1	1	∧	membrane	cell mobility and transcription regulation
ADML_HUMAN	0.279	0.371	0.593	0.4717	0.2515	0.0322	0	0	1	up	secreted	control of fluid and electrolyte homeostasis
AMPN_HUMAN	−0.294	−0.534	−1.471	0.7523	0.3260	0.0010	0	0	−1	down	cytoplasm	protease, regulation of angiogenesis
CALR_HUMAN	0.271	−0.040	−1.686	0.8717	0.9995	0.0012	0	0	−1	down	secreted	Calcium binding, ER function promotion
CBPE_HUMAN	0.481	0.586	0.620	0.0346	0.0080	0.0049	1	1	1	up	secreted	Protein C terminus processing
COF1_HUMAN	0.042	−0.334	−1.390	0.9981	0.5582	0.0001	0	0	−1	down	cytoplasm	cell morphology and cytoskeletal organization regulation
GCSH_HUMAN	−0.670	−0.832	−1.069	0.2004	0.0768	0.0196	0	0	−1	down	Mitochondrion	glycine degradation
HS90A_HUMAN	0.640	0.694	0.758	0.1053	0.0722	0.0446	0	0	1	up	cytoplasm	promote maturation and proper regulation of proteins
K1C10_HUMAN	−0.498	−0.766	−1.376	0.3749	0.0951	0.0011	0	0	−1	down	cytoplasm	structural consituent of epidermis
K22E_HUMAN	−0.382	−0.639	−1.797	0.7501	0.3867	0.0011	0	0	−1	down	keratin filament	keratinocyte activation, proliferation and keratinization
K2C1B_HUMAN	0.021	−0.138	−1.422	0.9999	0.9710	0.0026	0	0	−1	down	keratin filament	structural molecule activity
K2C1_HUMAN	−0.048	−0.404	−1.543	0.9991	0.6918	0.0034	0	0	−1	down	membrane	kinase activity regulation
LAMC1_HUMAN	0.118	−0.118	−1.047	0.9208	0.9208	0.0001	0	0	−1	down	secreted	mediate the attachment, migration and organization of cells into tissues
MK01_HUMAN	−0.714	−1.204	−1.693	0.4357	0.0716	0.0173	0	0	−1	down	cytoplasm	MAP kinase signal transduction pathway
PDIA1_HUMAN	0.149	−0.189	−1.058	0.9483	0.9029	0.0096	0	0	−1	down	Endoplasmic reticulum lumen	catalyzes the formation, breakage and rearrangement of disulfide bonds
PGAM2_HUMAN	0.028	0.021	0.987	0.9997	0.9999	0.0304	0	0	1	V	cytosol	interconversion of phosphoglycerate
PGS2_HUMAN	0.799	0.708	0.964	0.0773	0.1323	0.0263	0	0	1	V	secreted	affect the rate of fibrils formation
PLOD1_HUMAN	0.045	0.019	−1.092	0.9976	0.9998	0.0026	0	0	-1	down	Endoplasmic reticulum lumen	Forms hydroxylysine in collagens, promote stability of collagen cross-links
PROF1_HUMAN	0.131	−0.282	−1.113	0.9264	0.5841	0.0004	0	0	−1	down	cytoplasm	Binds to actin and affects the structure of the cytoskeleton
PROF2_HUMAN	−0.075	0.291	−1.088	0.9947	0.7876	0.0250	0	0	−1	∧	cytoplasm	Binds to actin and affects the structure of the cytoskeleton
PSME2_HUMAN	−−0.028	0.347	1.419	0.9999	0.8054	0.0177	0	0	1	up	cytosol	immunoproteasome assembly and efficient antigen processing
ROA2_HUMAN	−0.228	−0.264	−1.018	0.6391	0.5327	0.0002	0	0	−1	down	cytoplasm	pre−mRNA processing
SBSN_HUMAN	0.427	0.435	0.786	0.3140	0.2997	0.0221	0	0	1	up	secreted	process of epidermal differentiation
TCO1_HUMAN	−0.556	−0.483	−1.204	0.3644	0.4547	0.0107	0	0	−1	∧	secreted	binds to Vitamin B-12, transports cobalamin into cells
TPM1_HUMAN	0.477	0.520	0.634	0.1054	0.0700	0.0213	0	0	1	up	cytoplasm	Binds to actin filaments, regulation of muscle contraction
TPM4_HUMAN	0.532	0.613	0.586	0.0775	0.0355	0.0466	0	1	1	∧	cytoplasm	Binds to actin filaments, regulation of muscle contraction
TRFL_HUMAN	−0.208	−0.711	−1.154	0.7107	0.0120	0.0001	0	−1	−1	down	secreted	iron binding transport, antimicrobial activity
UBC9_HUMAN	−0.163	−0.278	−1.480	0.9320	0.7518	0.0010	0	0	−1	down	cytoplasm	SUMO ligation
UCHL1_HUMAN	0.416	0.552	0.601	0.1263	0.0289	0.0159	0	1	1	up	cytoplasm	Ubiquitin-protein hydrolase

Fold changes between treatment and sham groups were represented as group differences, only proteins with changes >1.5 fold (0.58 in log2 scale) for up- or <2.0 fold for down-regulation (−1 in log2 scale) were shown (full list of statistically significant proteins is shown in Supporting Information Table S1). Dunnet adjusted T-test was used to assess the statistical significance of the change. P values <0.05 were deemed statistically significant. Dunnet trend: 0, no significant change; 1, upregulation with statistical significance; −1, downregulation with statistical significance. Trend: general trend of the average abundance of proteins with increasing radiation dose; down, downregulation with increasing dose; up, upregulation with increasing dose; ∧, 10 cGy group had the highest abundance; V, 10 cGy group had the lowest abundance. Protein subcellular locations and biological functions were derived from Uniprot.

**Figure 2:**
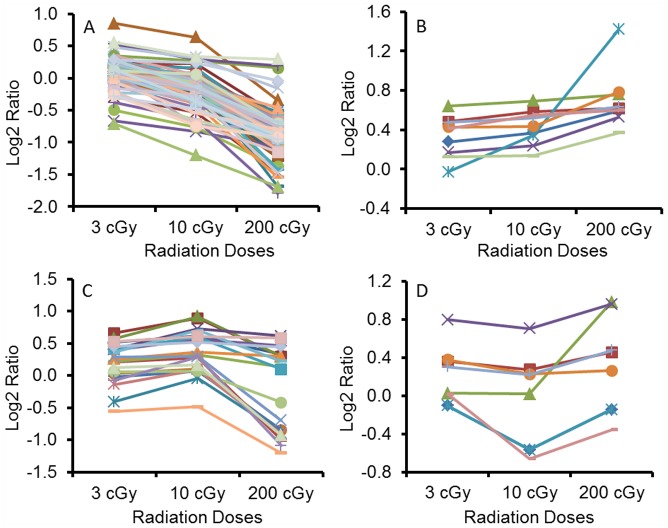
Trends associated with protein abundance profiles and doses of ionizing radiation. Protein abundance fold change at each radiation dose was normalized to that of sham (0 cGy) using log2 ratio and plotted as the y axis. Each line represents profile of one protein with increasing doses of ionizing radiation along the x axis. A) 97 proteins with their abundances decreased; B) 9 proteins with their abundances increased; C) 21 proteins with their abundances highest at 10 cGy; D) 8 proteins with their abundances lowest at 10 cGy. Detailed statistical analysis values, such as fold change and p values from Dunnett adjusted 2-sided t-test are listed in Supporting Information Table S1.

**Figure 3:**
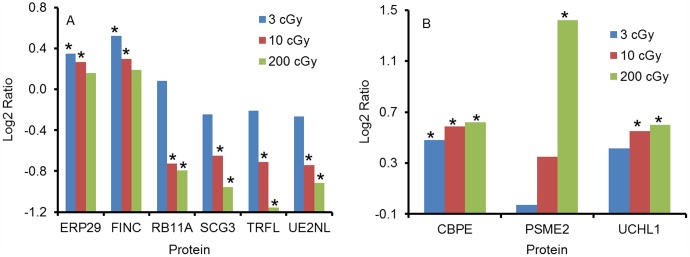
Representative proteins that showed A) consistent decrease and B) consistent increase with increasing radiation dose. The fold change of protein abundance at each dose was represented by the log2 ratio with respect to the value of sham group. * represents statistically significant (P<0.05, Dunnett adjusted 2 sided t-test). Detailed statistical analysis values, such as fold change and p values are listed in Supporting Information Table S1.

Among these 135 proteins, 97 proteins showed the trend of downregulation with increasing dose of X-rays, irrespective of whether they were more or less abundant when compared with the sham group (Figure 2A). A few families of proteins dominated this category, such as 14-3-3 family, keratin, collagen, alpha-actinin family, heat shock protein 70 family, etc. Although for 87 of these proteins their changes were only statistically significant (P<0.05) for the 200 cGy irradiated group when compared with the sham, there were 4 proteins, i.e. putative ubiquitin-conjugating enzyme E2 N-like (UE2NL), lactotransferrin (TRFL), secretogranin-3 (SCG3) and ras-related protein Rab-11A (RB11A) all showed significant downregulation for both the 10 and 200 cGy irradiated groups (Figure 3A). There were two proteins, endoplasmic reticulum resident protein 29 (ERP29) and fibronectin (FINC) both upregulated significantly for the 3 and 10 cGy irradiated groups (Figure 3A), but the general trend was still going down with increasing dose.

On the other hand, there were 9 proteins that showed the trend of upregulation with increasing radiation dose (Figure 2B), although for 7 of them the level of upregulation was only statistically significant upon exposure to 200 cGy of irradiation. Two proteins, carboxypeptidase E (CBPE) and ubiquitin carboxyl-terminal hydrolase isozyme L1(UCHL1) appeared to be sensitive to ionizing radiation, but relatively independent to doses received, as relatively little change was occurred between different dose groups. Conversely, proteasome activator complex subunit 2 (PSME2) protein was shown to be very sensitive to the dose of radiation, as rapid upregulation of this protein was observed when radiation doses were increased from 3, to 10 and 200 cGy (Figure 3B).

Unlike most of the proteins that have up or down changes associated with increasing radiation dose, there were 21 and 8 proteins being observed to have the largest change in the 10 cGy irradiated group, with the former one having the highest and the latter one the lowest level among all three radiated doses, respectively (Figure 2C&D). Although irregular, this pattern of change has been observed in the metabolomic profiling of the same 3D human skin tissue system exposed to X-ray irradiation as well.[7]

### Implications for interpreting the effects of exposure to ionizing radiation

The secretome is defined as the ensemble of proteins secreted into the extracellular space by a cell, tissue, organ, or organism at any given time and conditions through known and unknown secretory mechanisms involving constitutive and regulated secretory organelles. This term can be broadly defined to include the proteins of extracellular matrix (ECM) as well as additional proteins shed from the cell surface. Exosomes, due to their small size (< 100 nm), are technically challenging to be separated from culturing medium. Therefore, the secretome identified in our study may contain proteins released from exosomes as well. In addition to being masked by serum proteins (i.e. fetal bovine serum) that normally present in the culture media, for which we have applied immunodepletion for their removal, secretome of skin tissue could also be contaminated by non-secreted proteins released following cell lysis and death. In this respect, the levels of two major cytosolic proteins - beta-actin (ACTB_human) and beta-tubulin (TBB5_human) have been used by others to monitor cell autolysis.[12] Beta-tubulin was not detected in our study. Although beta-actin was detected, it didn’t appear to be abundant on the basis of the abundances of its constitutive peptides; in addition, our results did not show any significant changes of this protein within the medium samples collected from any of the irradiated groups, which demonstrated that the proteomic profile changes that we observed were reflective of the true skin secretome, not related to ionizing radiation-induced cell death/autolysis.

Certain forms of soluble keratin were reported to downregulate with high doses of X-ray irradiation,[6], [27] our results concurred their findings with the identification of four different forms of keratin - K1C10_HUMAN, K22E_HUMAN, K2C1B_HUMAN, K2C1_HUMAN all showed downregulation with respect to the sham controls, in particular the downregulation was statistically significant at the 200 cGy high dose (see Supporting Information Table S1). The 14-3-3 family is comprised of highly conserved proteins that are functionally important in the maintenance of homeostasis. They directly interact with various intracellular proteins and regulate a large spectrum of both general and specialized signalling pathways.[28] Their involvement with the cell cycle, association with proto-oncogenes and oncogenes, and abnormal expression in various tumors has linked this family of proteins to the etiology of human cancer.[29] In this respect, tumor suppressor protein 14-3-3-σ was reported to downregulate in a radioresistant nasopharyngeal cancer cell line when compared with regular nasopharyngeal cancer cell line,[30] and the same trend was observed for non-small cell lung cancer cells in comparison with normal human bronchial epithelial cells.[31] In addition, secreted 14-3-3 zeta has been demonstrated to have potential as candidate biomarkers for aggressive thyroid carcinomas.[32] The seven 14-3-3 proteins (see Supporting Information Table S1) observed in this work all showed downregulation for all of the irradiated samples, in particular for the 200 cGy dose when compared with the sham irradiation, which showed that ionizing radiation suppress expression of many proteins within the 14-3-3 family, this may lead to perturbed homeostasis in tumor suppression and other important biological functions.

High doses of ionizing radiation have suppressive effects to many biological processes. In this respect, it was reported that more downregulated proteins than upregulated proteins were being observed after tumor cells exposed to 10 Gy of X-rays, as a result of cell cycle suspension, suppression of cell proliferation and growth, and reduced metabolic processes from the effects of high doses of ionizing radiation.[33] Similarly, although measured at mRNA level, significant number (244 genes; 33%) of down regulated genes was observed when mammary gland tissues were exposed to 2 Gy of ionizing radiation; in particular, a number of genes with tumor suppressor function persistently downregulated in response to radiation exposure.[34] Consistent with these reports, majority (102 of 135, Supporting Information Table S1) of the significantly changed proteins observed in this study showed statistically significant downregulation for the 200 cGy high dose. Ingenuity pathway analysis results showed that most of these proteins were associated with organization of extracellular matrix, cell signalling, cell migration, cell proliferation and apoptosis.

On the other hand, despite the general downward trend line of protein expression with the increase of radiation dose as observed in Figure 2A, many of the proteins appeared to be upregulated in comparison with sham controls at 3 and 10 cGy, although changes at these two low doses were largely not statistically significant compared to sham controls. This implies that low doses of ionizing radiation likely have stimulating effects on the secretome, which is in agreement with proteomic studies of endothelial cell exposure to low dose gamma radiation (<50 cGy).[35], [36]

## Conclusion

To our knowledge, this work represents the first global proteomic profiling and quantitative comparison of the *in vitro* skin tissue secretome after exposure to ionizing radiation. While it is consistent with the previous studies that the majority of the secreted proteins were downregulated as a result of high dose (2 Gy) ionizing radiation-induced stress and damage, due to decreased metabolic processes and suppressed cell cycle and growth; many of these secreted proteins at low doses were upregulated compared with sham controls. Since it is unclear whether this response is specific to ionizing radiation or represents a general response to injury, the proteins that displayed unique patterns of regulation, or being sensitive to either ionizing radiation or dose level, warrant further study for their roles in adaptive responses of ionizing radiation, and their robustness as biodosimetry markers when combined with other agents that may induce similar damaging effects.[37], [38]

### 

#### Data Deposition:

All data related to this work were deposited in PeptideAtlas, as in http://www.peptideatlas.org/PASS/PASS00404 and http://www.peptideatlas.org/PASS/PASS00405.

## Supporting Information

Table S1 **Statistically significant proteins secreted into the medium by a full thickness skin tissue model at different doses (3, 10 and 200 cGy) of X-rays when compared with sham controls (0 cGy).** Raw data of protein abundance were log2 transformed, as represented by the average of each irradiated group. Fold changes between treatment and sham groups were represented as group differences. Dunnet adjusted T-test was used to assess the statistical significance of the change. P values <0.05 were deemed statistically significant. Dunnet trend: 0, no significant change; 1, upregulation with statistical significance; -1, downregulation with statistical significance. Trend: general trend of the average abundance of proteins with increasing radiation dose; down, downregulation with increasing dose; up, upregulation with increasing dose; ∧, 10 cGy group had the highest abundance; V, 10 cGy group had the lowest abundance. Protein name and their subcellular location were derived from Uniprot.
(XLSX)
